# Classification of Magnetic Nanoparticle Systems—Synthesis, Standardization and Analysis Methods in the NanoMag Project

**DOI:** 10.3390/ijms160920308

**Published:** 2015-08-27

**Authors:** Sara Bogren, Andrea Fornara, Frank Ludwig, Maria del Puerto Morales, Uwe Steinhoff, Mikkel Fougt Hansen, Olga Kazakova, Christer Johansson

**Affiliations:** 1 Acreo Swedish ICT AB, Arvid Hedvalls Backe 4, Box 53071, SE-400 14 Göteborg, Sweden; E-Mail: sara.bogren@acreo.se; 2 SP Technical Research Institute of Sweden, Box 5607, SE-114 86 Stockholm, Sweden; E-Mail: andrea.fornara@sp.se; 3 Institute of Electrical Measurement and Fundamental Electrical Engineering, TU Braunschweig D-38106, Germany; E-Mail: f.ludwig@tu-bs.de; 4 Instituto de Ciencia de Materiales de Madrid, ICMM-CSIC, Cantoblanco, 28049 Madrid, Spain; E-Mail: puerto@icmm.csic.es; 5 Physikalisch-Technische Bundesanstalt, D-10587 Berlin, Germany; E-Mail: Uwe.Steinhoff@ptb.de; 6 Department of Micro and Nanotechnology, Technical University of Denmark, DTU Nanotech, Building 345 East, Kgs. Lyngby DK-2800, Denmark; E-Mail: mikkel.hansen@nanotech.dtu.dk; 7 National Physical Laboratory, TW11 0LW Teddington, UK; E-Mail: olga.kazakova@npl.co.uk

**Keywords:** magnetic nanoparticles, nanostructures, standardization, magnetic synthesis, magnetic analysis, magnetic material

## Abstract

This study presents classification of different magnetic single- and multi-core particle systems using their measured dynamic magnetic properties together with their nanocrystal and particle sizes. The dynamic magnetic properties are measured with AC (dynamical) susceptometry and magnetorelaxometry and the size parameters are determined from electron microscopy and dynamic light scattering. Using these methods, we also show that the nanocrystal size and particle morphology determines the dynamic magnetic properties for both single- and multi-core particles. The presented results are obtained from the four year EU NMP FP7 project, NanoMag, which is focused on standardization of analysis methods for magnetic nanoparticles.

## 1. Introduction

**Figure 1:**
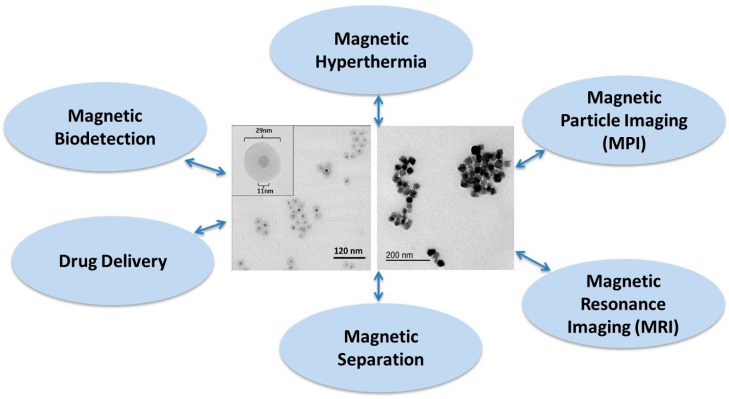
Biomedical applications of magnetic nanoparticles. Both single- and multi-core magnetic nanoparticle systems as shown in the TEM images are used in the applications.

Single-core magnetic iron oxide nanoparticles with sizes from a few nanometers and iron oxide based multi-core particles with sizes up to several micrometers can be found in several biomedical applications in the areas of diagnosis, therapy, actuation and imaging [1,2]. Magnetic nanoparticle systems can act as binding sites and nanosensors in magnetic biosensor detection systems, they can be local heat sources in magnetic hyperthermia to kill cancerous cells, they facilitate separation steps in immunoassays, they can act as drug carriers in targeting procedures or they can act as imaging agents in magnetic resonance imaging or magnetic particle imaging [1,2]. In many of these biomedical applications, the parameters of the particle size distribution, both for the single- and multi-core particles, are important to know and to control during the synthesis process. Some biomedical applications using magnetic nanoparticles (MNPs) are illustrated in Figure 1.

MNPs can be investigated in immobilized state or suspended in a liquid. The latter is relevant for many biomedical applications. Measurements on immobilized MNPs provide important information on structural and magnetic properties of the metal oxide MNP cores. Immobilization of MNPs can be reached in several ways: air drying, freeze drying, embedding in gel, sugar or gypsum, or by other methods. It has been observed that the magnetic behavior of immobilized samples depends on the actual method of immobilization, which will alter the local environment and organization of the MNPs. Nevertheless, the interpretation of measurement data of immobilized MNP can be based on well-established models. The ensemble behavior of immobilized MNPs, in most cases, does not represent the suspension behavior, firstly because the interaction of the particles and the suspension medium is missing and secondly, because the average separation and thus the dipolar interaction energy between the particles have changed. MNP suspensions, on the other hand, make up realistic samples for the ensemble behavior, but the model-based interpretation might become highly complicated due to field-dependent changes in MNP organization and interactions (e.g., chain formation), which have to be included in the interpretation of measurement data. These effects may further depend on the degree of dilution of the MNPs in the suspension medium, which should also be taken into account.

**Figure 2:**
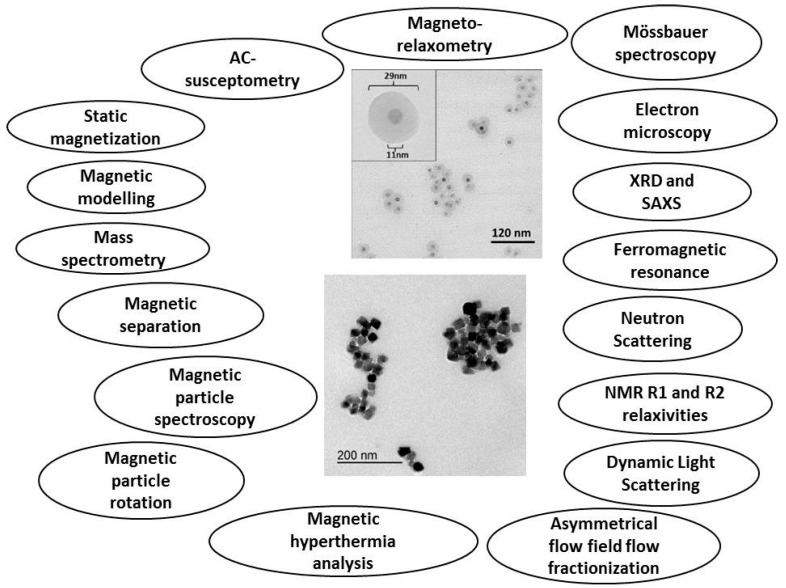
Analysis techniques used in the NanoMag project for the characterization and analysis of synthesized single- and multi-core nanoparticles shown in the TEM (transmission electron microscopy) images in the middle of the figure.

Although MNPs have been researched and applied already for a considerable number of years, so far there exists no standardized way of characterizing and expressing their main physicochemical properties. This represents a considerable obstacle for introducing new MNP-based biomedical and clinical applications, for example Magnetic Particle Imaging (MPI). Within the EU FP7 project, NanoMag, as a possible means to improve this situation, we have formulated a tentative normative document for a standardized MNP description. This document will then iteratively be rewritten and updated, in strong collaboration with MNP manufacturers, appliers, end-users and other socioeconomic groups interested in MNPs. Eventually, the knowledge and result obtained in the NanoMag project can enter a standardization process as it is mediated by the European Committee for Standardization and the European Committee for Electrotechnical Standardization (CEN/CENELEC) or the International Organization for Standardization (ISO). A stakeholder committee consisting of 15 industrial, medical and academic organizations guides the NanoMag project regarding standardization and exploitation. The four-year NanoMag project focused on nanometrology standardization methods for MNPs was launched in November 2013. The project involves research institutes, universities, companies as well as national metrology institutes. The main objectives of this project are to improve and redefine existing analysis methods and models and to develop new standardized analysis methods and models for MNPs. Using improved manufacturing technologies, the NanoMag project will synthesize MNPs with specific properties, characterize them with a multitude of techniques (focusing on both structural and magnetic properties), and bring the experimental results together to obtain a self-consistent picture, which describes, e.g., how structural and magnetic properties are interrelated. This extensive project aims to define the standard measurements and techniques required to classify and characterize magnetic nanoparticle systems and to be used for quality control during synthesis of magnetic nanoparticles. A detailed description of the project can be found on the NanoMag website [3]. In the NanoMag project, we use basic analysis and more advanced analysis techniques as well as more application oriented methods, summarized in Figure 2. A detailed description of the analysis methods for MNP systems is available now and allows the selection of a tailored analysis strategy depending on target properties, economical effort other application aspects.

MNPs synthesized within the project as well as selected commercially available nanoparticles are characterized and analyzed with the techniques and methods that are shown in Figure 2. The results are used to identify and standardize analysis methods for MNPs. All of the analysis methods shown in Figure 2 have been used in the first two years of the NanoMag project.

**Figure 3:**
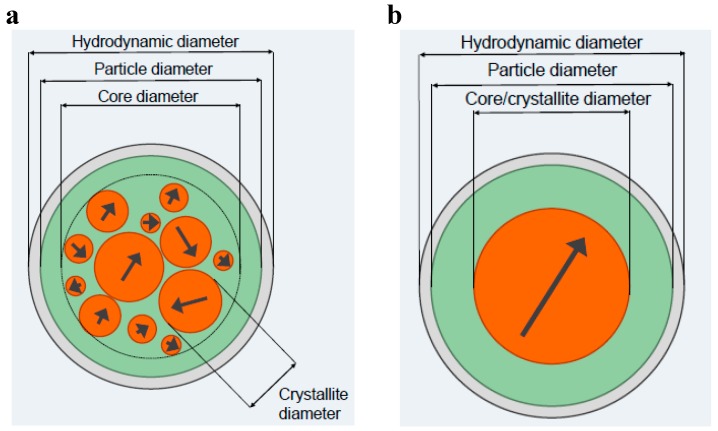
Schematic pictures of magnetic (**a**) multi-core particles and (**b**) single-core particles. The definitions of the size parameters of the two different particle types are also shown in the figure. See the text for an explanation of the compartments.

Since the behaviour of MNPs in a suspension crucially depends on their physical properties (for instance, the mean size and the distribution of sizes of both the nanocrystals and the physical particles as well as the hydrodynamic size and surface charge that determine the colloidal stability) and the type of MNP systems (single- or multi-core particle system), it is of vital importance for different biomedical applications that the analysis methods used to determine the physical parameters are well defined and standardized. In the description of the structural composition of a typical iron oxide based MNP suspension, there should be a clear distinction between the different compartments of the suspension: metal oxide cores, organic coating and suspension medium (see Figure 3). A single-core magnetic nanoparticle can be defined as a particle containing only a single nanocrystal that can be further coated and functionalized at the surface for colloidal stability and also for use in specific applications (e.g., specific binding to different analytes). A multi-core particle can be defined as a particle containing several nanocrystals either densely or loosely packed within the multi-core structure. The surface of the multi-core particle can also be coated and functionalized. We have earlier presented a review of synthesis of single-core and multi-core particles [4]. The magnetic properties of single-core and multi-core particles depend on both the properties of the nanocrystals (such as their size distribution) and in the case of multi-core particles how the nanocrystals are distributed in the multi-core structure [5,6]. Schematic pictures of single- and multi-core particles are shown in Figure 3.

The particle size parameters for a multi-core particle system can be defined according to their (I) nanocrystal size (crystallite diameter); (II) the core diameter where the nanocrystals are positioned; (III) the particle diameter due to the particle matrix (for instance starch or dextran) and surface coating and/or surface functionalization; and (IV) the hydrodynamic diameter if the particles are dispersed in a carrier liquid (for instance water), which may be different from the physical particle size due to the solvation zone near the particle surface that is dragged along with the particle when it rotates. For the single-core particle only one single-domain nanocrystal is within the particle and the defined particle size parameters are the same as for the multi-core particle except that the core size is the same as the nanocrystal size.

Magnetic relaxation in a MNP system dispersed in a carrier liquid can be divided in particles that undergo internal Néel relaxation where the magnetic moment within the nanocrystals rotates due to thermal activation and is decoupled from the particle rotation. The other relaxation mechanism is the Brownian relaxation where the particle physically rotates and the magnetic moments of the nanocrystals are blocked in a specific direction in the particles and the total effective particle magnetic moment is coupled to the particle and rotates with the same rate as the particle itself [5]. Both processes may take place simultaneously. However, the faster process will dominate the overall magnetic relaxation behavior of the particle system.

MNP systems (both single- and multi-core nanoparticles) may contain small nanocrystals that show fast internal magnetic relaxation (Néel relaxation) and larger nanocrystals that are thermally blocked. An ensemble of single-core particles, with a typical relaxation time shorter than the specific time scale of the measurement (for instance defined by the measurement excitation frequency in AC susceptometry measurements), behaves as a superparamagnetic material and shows a magnetic response in phase with the applied magnetic field. When the time scale of the measurement is of the same order of magnitude as the relaxation time, the magnetic response of the particle ensemble will lag behind the magnetic field excitation and have a non-zero out-of-phase component.

The Néel relaxation time in zero magnetic field and assuming no magnetic interactions between the nanocrystals is:

(1) $${\text{τ}}_{N}={\text{τ}}_{0}\text{exp}\left(\frac{KV_{C}}{kT}\right)$$

where τ_0_ is the intrinsic relaxation time of the magnetic nanocrystals, *K* the magnetic anisotropy, *V_C_* the volume of the nanocrystals, *k* the Boltzmann constant and *T* is the temperature.

The Brownian relaxation time is:

(2) $${\text{τ}}_{B}=\frac{3V_{H}\text{η}}{kT}$$

where *V_H_* is the hydrodynamic volume (given by 4π*r_H_*^3^/3, where *r_H_* is the hydrodynamic radius) of the magnetic nanoparticles (taking into account the particle surface layer and the volume of carrier liquid the particles drags along when rotating) of the particles and η is the viscosity of the carrier liquid in which the particles are dispersed. The total relaxation time, τ_eff_, the magnetic nanoparticles will undergo is given by the effective relaxation time combining both the Néel and Brownian relaxation time according to:

(3) $$\frac{1}{{\text{τ}}_{\text{eff}}}=\frac{1}{{\text{τ}}_{N}}+\frac{1}{{\text{τ}}_{B}}$$

**Figure 4:**
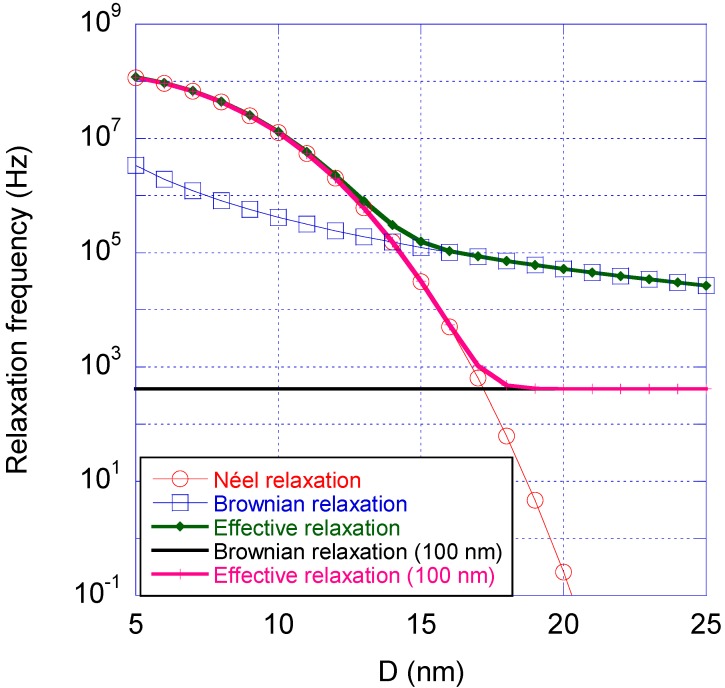
Magnetic relaxation time *vs.* the nanocrystal size (*D* is the diameter of the nanocrystals) for an iron oxide based nano-sized particle system dispersed in water at room temperature, showing the Néel relaxation frequency (red), Brownian relaxation frequency (blue) and the effective relaxation frequency (green). The figure also shows the Brownian relaxation frequency of a particle with diameter 100 nm (black) and the effective relaxation (magenta) a nanocrystal will undergo positioned in that particle matrix. The parameters of nano-sized iron oxide nanocrystals used in this figure were *K* = 20 kJ/m^3^, τ_0_ = 10^−9^ s, η = 10^−3^ Pa·s and zero shell thickness of the nanocrystals.

A corresponding relaxation frequency *f*_R_ can be defined as

(4) $$f_{\text{R}}=\frac{1}{2\text{πτ}}$$

where τ can be the Néel relaxation time (Equation (1)), Brownian relaxation time (Equation (2)) or the effective relaxation time (Equation (3)). The relaxation frequency, *f*_R_, can approximately be seen in the AC susceptibility *vs.* excitation frequency, *i.e.*, AC susceptometry (ACS) response in the same frequency range as the peak in the out of phase component and a decrease in the in-phase component. Figure 4 shows calculated values of the Néel, Brownian and effective relaxation frequencies *vs.* nanocrystal diameter for a single-core particle with a physical size equal to the magnetic size and a physical size equal to 100 nm, respectively. The parameters determining whether Néel or Brownian relaxation dominates for a magnetic nanoparticle system dispersed in a carrier liquid at a given temperature, are the mean sizes, size distribution, the magnetic material properties (through the magnetic anisotropy) and the viscous properties of the liquid (through the viscosity of the carrier liquid). At a specific temperature, the Néel relaxation depends mainly on the intrinsic properties of the nanocrystals (size and magnetic anisotropy) whereas the Brownian relaxation depends mainly external properties (carrier liquid viscosity). Both the Néel and Brownian relaxation mechanisms are affected by nanocrystal interactions and by the magnitude of the applied external measurement field (which is not taken into account in Figure 4).

Figure 4 shows that small nanocrystals relax via the Néel process, whereas larger nanocrystals relax via the Brownian process. The crossover size is in the case shown in Figure 4 is about 15 nm. If instead the nanocrystals are placed in a particle matrix with diameter of 100 nm the crossover size between Néel and Brownian relaxation is about 18 nm, see Figure 4.

Dynamic magnetic properties of magnetic nanoparticle systems can be analyzed by using ACS (AC susceptometry) analysis and MRX (magnetorelaxometry) analysis (magnetic moment *vs.* time after switching off the magnetizing field). Several earlier studies using ACS and MRX analysis for determining dynamic magnetic properties and particle size parameters in MNP systems has been carried out [5,6,7,8,9,10,11,12,13,14,15].

The frequency dependent complex magnetic AC susceptibility can be described by the in-phase component, χ′, (real part) and the out-of-phase component, χ′′, (imaginary part) according to:

(5) $$\text{χ}={\text{χ}}^{\prime }-i\chi^{\prime \prime }$$

In order to extract MNP parameters from the AC susceptometry data we use ACS models depending on the class of particle system investigated.

AC susceptibility model for single-core particles is described by the following relation [7]:

(6) $$\text{χ}\left(\text{ω}\right)=C\int^{\text{​}}\frac{r_{C}^{6}}{\left(1+i{\text{ωτ}}_{\text{eff}}(r_{C},\text{δ}\right)}f(r_{C})dr_{C}+{\text{χ}}_{\text{high}}$$

*f*(*r*_C_) is the number-weighted nanocrystal size distribution (log-normal function is used), *r^H^* = *r^C^* + δ where *r_C_* is the magnetic nanocrystal radius,, ω = 2π*f* where *f* is the excitation frequency and *C* is a pre-factor (including temperature, intrinsic saturation magnetization and particle density), δ is the thickness of the shell surrounding the nanocrystals, and χ_high_ is the high frequency value of the in-phase part of the susceptibility. This high frequency relaxation process is probably due to the intra-potential-well contribution of the nanocrystals to the AC susceptibility [5,7].

The multi-core model is used for multi-core structured particles that undergo Brownian relaxation. Since the nanocrystals in the multi-core can magnetically interact with each other, non-interacting models for the (I) dynamic AC response and (II) the Néel relaxation time cannot be used. Instead we use a mean value for the DC (static) susceptibility, *<*χ_0*B*_>, in the Debye model. The mean value of <χ_0*B*_> is then the average DC susceptibility value of the multi-core particle system. Thus, we picture each multi-core particle as a magnetic site with a mean value susceptibility <χ_0*B*_>. Since we use a mean value of the susceptibility, <χ_0*B*_>, we make the approximation that all multi-core particles contributes equally to the DC susceptibility. The AC susceptibility response can then be expressed as [8]:

(7) $$\text{χ}(\text{ω})\;=\int \frac{{\text{χ}}_{0B}\left(r_{H}\right)}{\left(1+i{\text{ωτ}}_{B}\left(r_{H}\right)\right)}f\left(r_{H}\right)dr_{H}+{\text{χ}}_{\text{high}}={\text{<χ}}_{\text{0B}}\text{>}\int \frac{1}{\left(1+i{\text{ωτ}}_{B}\left(r_{H}\right)\right)}f\left(r_{H}\right)dr_{H}+{\text{χ}}_{\text{high}}$$

where *f*(*r_H_*) is the hydrodynamic particle size distribution (log-normal function is used). It has been shown in many earlier studies that using the above model that the determined hydrodynamic size distribution and mean particle sizes by fitting data to Equation (7), resembles very well the intensity weighed size distribution and the Z-average size as determined by DLS analysis [5,6,7,9].

Using the same approximation as described in connection to Equation (7) for multi-core particle systems that shows a mixture of both Brownian and Néel relaxation, the AC susceptibility model for multi-core particles (Equation (7)) is combined with a Cole–Cole expression for the Néel relaxation part, according to [8]:

(8) $$\text{χ}\left(\text{ω}\right)={\text{χ}}_{0_{B}}\;\int \frac{1}{\left(1+i{\text{ωτ}}_{B}\left(r_{H}\right)\right)}f\left(r_{H}\right)dr_{H}+\frac{{\text{χ}}_{0_{N}}}{1+{\left(i{\text{ωτ}}_{N}\right)}^{\alpha }}+{\text{χ}}_{\text{high}}$$

where χ_0*N*_ gives the Néel DC susceptibility contribution, and α is the Cole–Cole parameter for the Néel relaxation part (0 < α < 1) that sets the width of the relaxation distribution. In addition, also in this case, it has been shown in earlier studies that using the above equation that the determined size distribution resembles very well the intensity weighed size distribution as determined by dynamic light scattering analysis.

The models given in Equations (6)–(8) are used to extract magnetic nanoparticle properties by fitting the models to the experimental data. The results for different particle types are given in chapter 2, where magnetic nanoparticle systems are classified according to their magnetic relaxation properties and their particle size parameters. Other similar AC susceptometry models can be used in order to extract size parameters of magnetic nanoparticle systems [10,11]. Models are also used to analyse the MRX data in order to extract for instance particle size parameters [12,13,14].

We are not able to, in a single article, present the result from all analyzing techniques in the NanoMag project. In this article, we will concentrate on the result regarding some synthesis of single- and multi-core nanoparticles in the NanoMag project and how to classify these particle systems, by analysing their magnetic relaxation properties using ACS and also taking into account their nanocrystal size and particle size and morphology using transmission electron microscopy (TEM) and dynamic light scattering (DLS) data. MRX analysis is also used as complementary magnetic relaxation measuring technique in order to verify the ACS relaxation measurements presented in chapter 2. TEM analysis gives the number weighted size distribution, while the DLS analysis gives primarily the intensity weighted (∝$r_{H}^{6}$) size distribution (and Z-average size). When comparing the results of the determined mean sizes from each analysis technique for each of the particle systems, as given in chapter 2, we take into account the different weighting of the size distribution for the different measuring techniques.

## 2. Results and Discussion

In this chapter, we will describe and give examples of how single-core and multi-core MNP systems dispersed in a carrier liquid (for instance water), which can be classified regarding their magnetic relaxation properties determined from ACS and MRX and their particle size parameters as determined from TEM and DLS (hydrodynamic size in solution contributing particle plus coating).

### 2.1. Single-Core Particle System with Néel Relaxation

When the Néel relaxation of the nanocrystals dominates (*i.e.*, the Néel relaxation is faster than the Brownian relaxation), the effective magnetic moment of a particle is decoupled from the physical orientation of the particle.

**Figure 5:**
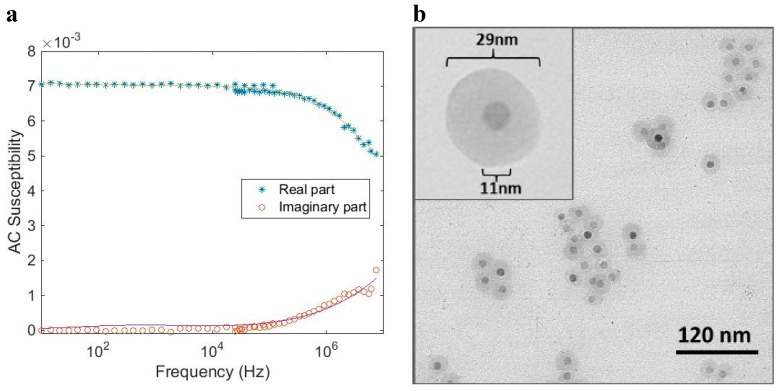
(**a**) AC (dynamical) susceptibility *vs.* frequency at room temperature of a magnetic single-core particle system (*CSIC01*) that shows Néel relaxation (particles dispersed in water). The solid line in the figure is from the fitting procedure to the single-core model; (**b**) TEM image of the particle system.

Figure 5 shows AC susceptibility measurements and a TEM image of a single-core particle system (*CSIC01*) that undergoes Néel relaxation. The Néel relaxation peak in the out of phase component is in the range of 10 MHz. The TEM image (Figure 5b) shows that the particles consist of an iron oxide nanocrystal with a mean nanocrystal size of 11 nm encapsulated in a silicon dioxide shell to form a particle with a total mean diameter of 29 nm. From the TEM image, we can also see that some of the magnetic single-core particles have clustered. This is attributed to the preparation of the TEM sample. An analysis of the AC susceptibility data measured *vs*. frequency in Figure 5a in terms of Equation (6), assuming Néel relaxation with *K* = 20 kJ/m^3^, yields a nanocrystal core size in the range of 11 nm, consistent with the TEM analysis. MRX measurements on particles in a liquid carrier medium (deionized water in this case) as well as in a solid matrix confirm that Néel relaxation dominates in this particle system. Analysis by DLS resulted in a hydrodynamic diameter of 91 nm (Z-average size) indicating that some agglomeration of the coated particles had taken place, although an overestimation in DLS size due to intensity weighted statistics being dominated by a few large particles cannot be ruled out, since the obtained size distributions from the TEM images are number weighted. As a comparison, we can transform the DLS Z-average size to a particle number weighed size distribution and obtain a mean particle size of 54 nm. As seen in Figure 4, this particle system, with a nanocrystal size of 11 nm, shall undergo Néel relaxation. This is in agreement with the results of the ACS analysis.

### 2.2. Single-Core Particle System with Brownian Relaxation

When the Brownian relaxation dominates (*i.e.*, the Néel relaxation time is longer than the Brownian relaxation time), the effective magnetic moment of a particle is linked to the physical orientation of the particle. In this case, the effective magnetic moment of the particle rotates with the same rate as the particle itself and the relaxation frequency is given by the Brownian relaxation frequency.

**Figure 6:**
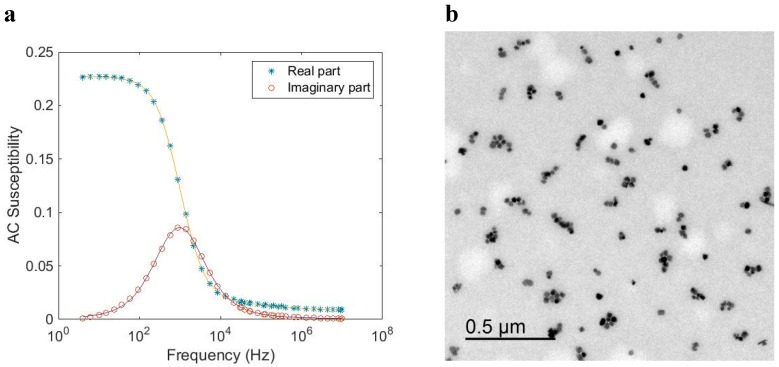
(**a**) AC susceptibility *vs.* frequency at room temperature of a magnetic single-core particle system (*CSIC04*) that shows Brownian relaxation (particles dispersed in water). The solid line in the figure is from the fitting procedure to the single-core model; (**b**) TEM image of the particle system.

**Figure 7:**
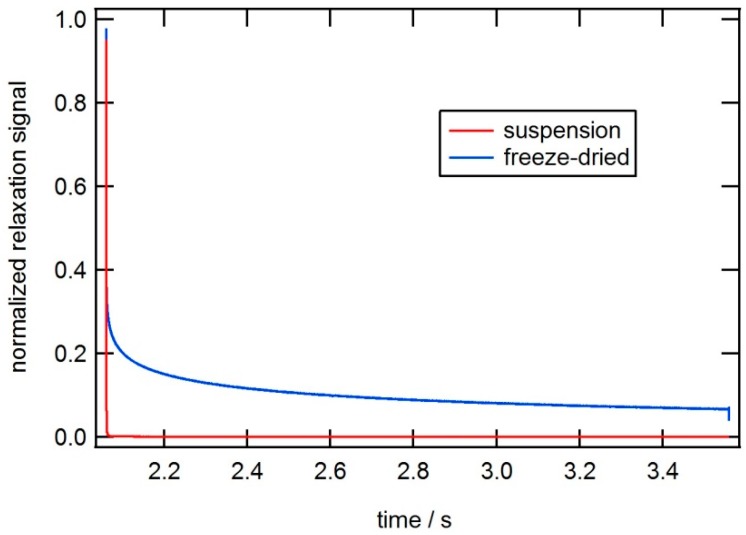
MRX (magnetorelaxometry) data at room temperature of a magnetic single-core particle system (*CSIC04*) that shows Brownian relaxation when particles are suspended in liquid (in red), and long Néel relaxation when the particles are immobilized (in blue).

Figure 6 shows AC susceptibility measurements and a TEM image of a single core particle system (*CSIC04*) with a larger size of the core crystals as compared with the previous particle system. In this case, the particles are coated with dextran. The Brownian relaxation peak in the out-of-phase component of the AC susceptibility is about 900 Hz. From the TEM image (Figure 6b), we determine a mean nanocrystal size of 25 nm, and, from the ACS analysis, we determine a mean nanocrystal diameter of 27 nm, which is in good agreement with TEM data. From the TEM image we also observed that some of the magnetic single-core particles have clustered. This attributed to the preparation of the TEM sample. MRX measurements on particles in a solid matrix that immobilizes the particles and suppresses the Brownian relaxation, show a long Néel relaxation time and confirm that Brownian relaxation is dominating when these particles are dispersed in a carrier liquid (for instance water as in this case) (see Figure 7).

Analysis of the AC susceptibility data in terms of Brownian relaxation yielded a mean hydrodynamic diameter of 77 nm, which is in agreement with the value 72 nm as obtained by DLS (Z-average size). According to Figure 4, this particle system with a nanocrystal size of 25 nm shall undergo Brownian relaxation. This is in agreement with the results of the ACS and MRX analysis.

### 2.3. Multi-Core Particle System with Néel Relaxation

When the Néel relaxation of the nanocrystals in the multi-core particle structure is faster than the Brownian relaxation, the effective magnetic moment of a particle is decoupled from the physical orientation of the particle. In this case, the relaxation frequency will be high even if the nanocrystals are positioned in a multi-core structure with a larger particle size.

**Figure 8:**
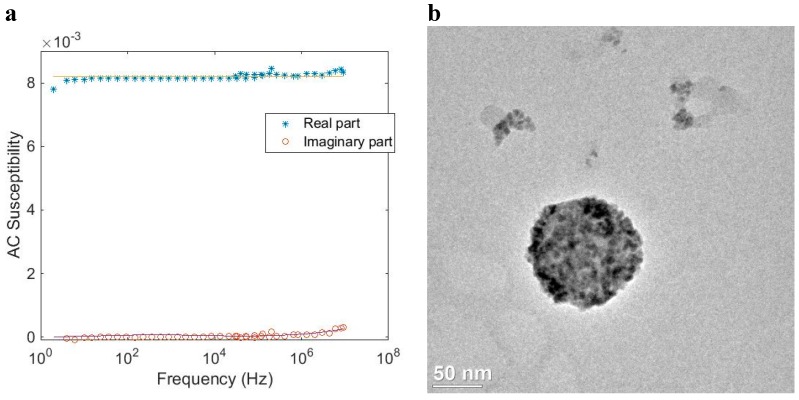
(**a**) AC susceptibility *vs.* frequency at room temperature of a magnetic multi-core particle system (*SP02*) that shows fast Néel relaxation; (**b**) TEM image of the particle system.

Figure 8 shows AC susceptibility measurements and a TEM image of such a system, where iron oxide nanocrystals are embedded in a matrix of polystyrene/poly(styrene-alt-maleic acid). The Néel relaxation peak in the out of phase component of the out of phase component is above 10 MHz. From TEM images, we determine a mean nanocrystal size of 8.7 nm and a mean multi-core particle size of about 54 nm. Figure 8b shows a close up TEM image of one of the multi-core particles. The hydrodynamic size of the particles determined by DLS is 130 nm (Z-average size). The discrepancy between the particle sizes determined by TEM and DLS may be due to agglomeration and/or the fact that the particles are dispersed in water and hence may have a hydrodynamic size, which is larger than the physical particle size. However, an overestimation in DLS size due to intensity weighted statistics being dominated by a few large particles cannot be ruled out, since the obtained size distributions from the TEM images are number weighted. From the AC susceptibility measurements (Figure 8a), assuming Néel relaxation and comparison with previous results, we obtain a nanocrystal size in the range below 10 nm. Since the relaxation peak is not fully developed in the ACS data, the nanocrystals size can only roughly be determined. According to figure 4 this particle system with a nanocrystals size of 8.7 nm shall undergo Néel relaxation. This is in agreement with the results of the ACS analysis. Accordingly, MRX curves both on suspended and immobilized particles show that the magnetic moments can follow the field pulse almost immediately (*i.e.*, the particle dynamic magnetization relaxes fast compared to the experimental time scale).

### 2.4. Multi-Core Particle System with Brownian Relaxation

When the Néel relaxation of the nanocrystals in the multi-core structure is longer than the Brownian relaxation, the effective magnetic moment of the particles are coupled to the physical rotation of the particles. In this case, the effective magnetic moment of the particles rotates with the same rate as the particle itself and the relaxation frequency is given by the Brownian relaxation frequency.

**Figure 9:**
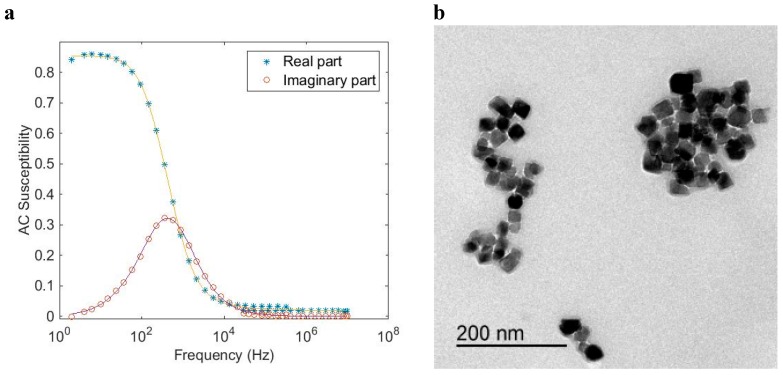
(**a**) AC susceptibility *vs.* frequency at room temperature of a magnetic multi-core particle system (*CSIC05*) that shows Brownian relaxation with a relaxation frequency at about 300 Hz. The solid line in the figure is from the fitting procedure to the multi-core model; (**b**) TEM image of the particle system.

**Figure 10:**
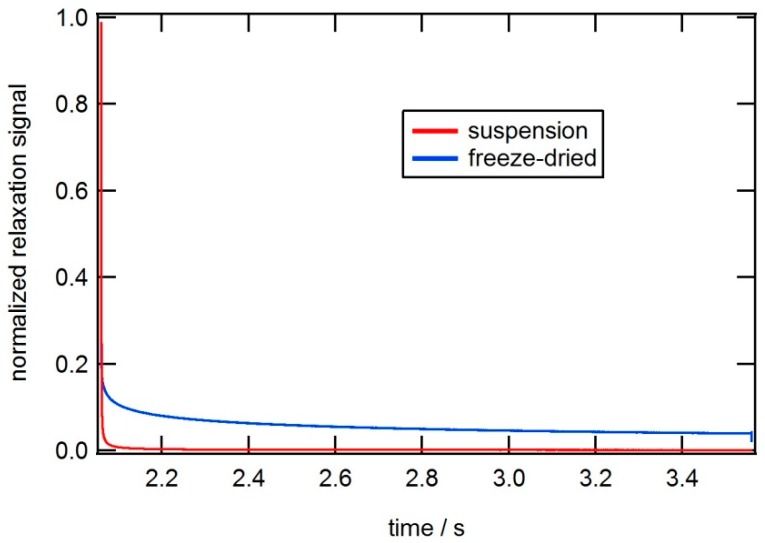
MRX data at room temperature of a magnetic multi-core particle system (*CSIC05*) that shows Brownian relaxation when particles are suspended in liquid (in red), and long Néel relaxation when the particles are immobilized (in blue).

Figure 9 shows AC susceptibility measurements and a TEM image of a multi-core particle system (*CSIC05*), where iron oxide nanocrystals showing blocked magnetic behavior are embedded in a matrix of dextran. The Brownian relaxation peak is about 300 Hz. From TEM images (close up TEM image shown in Figure 9b), we determine a mean nanocrystal size of 28 nm. The multi-core particles are of irregular size and range in sizes between 50 and 200 nm. From DLS analysis, we obtain a hydrodynamic diameter of 108 nm (Z-average size). From analysis of the AC susceptibility data (Figure 9a) assuming Brownian relaxation, we obtain a mean hydrodynamic particle size of 100 nm. Fitting MRX measurements on the suspended and immobilized particles with the moment superposition model [12] provide a mean hydrodynamic diameter of 108 nm and a mean core diameter of 28 nm, respectively. In Figure 10, the result from the MRX measurements can be seen.

The particle sizes obtained by ACS, MRX and DLS are again in good agreement. According to Figure 4 this particle system with a nanocrystals size of 28 nm shall undergo Brownian relaxation. This is in agreement with the results of the ACS and MRX analysis.

### 2.5. Mixed Particle Systems

If the MNP system has contributions from both Néel and Brownian relaxation, it will be observed as two peaks in the magnetic relaxation spectrum, where the peak at lower frequencies is attributed to Brownian relaxation and that at higher frequencies is attributed to Néel relaxation.

**Figure 11:**
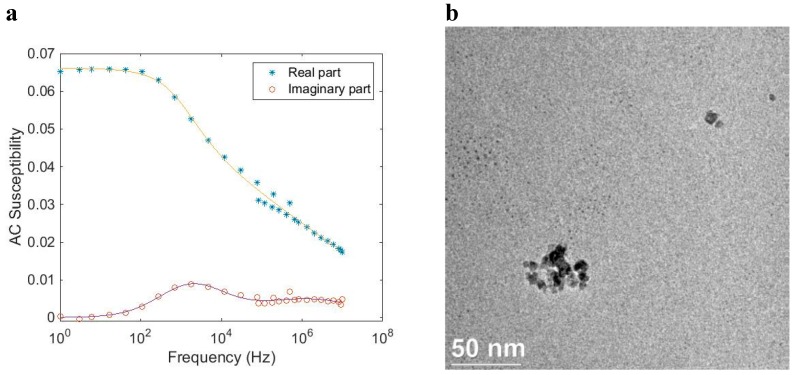
(**a**) AC susceptibility *vs.* frequency at room temperature of a magnetic nanoparticle system (*NPG3311*) that exhibits a mixture of Brownian and Néel relaxation. The solid line in the figure is from the fitting procedure to the extended multi-core model; (**b**) TEM image of the particle system. Nanocrystals not belonging to a multi-core structure (e.g., upper right in image) are likely to give the Néel response signal.

Figure 11 shows AC susceptibility measurements and a TEM image of a multi-core system, where iron oxide nanocrystals showing both blocked magnetic behavior with a Brownian relaxation at lower frequencies (in the range of 1 kHz) and a broad Néel relaxation at higher frequencies above 10 kHz. From the TEM image (Figure 11b), we determine a mean nanocrystal size of 11 nm and a mean multi-core particle size of 41 nm. From DLS analysis, we obtain a hydrodynamic diameter of 80 nm (Z-average size). From analysis of the AC susceptibility data (Figure 11a) assuming a mixture between Brownian and Néel relaxation, we obtain a mean hydrodynamic particle size of the Brownian relaxation of 78 nm, in good agreement with DLS data The particle sizes obtained by ACS and DLS are again in good agreement. According to Figure 4, this particle system with a nanocrystals size of 11 nm shall undergo only Néel relaxation. According to the ACS analysis, we also see the Brownian relaxation at lower frequencies, meaning that probably the nanocrystals in the multi-core structure magnetically interacts resulting in a blocking of the individual nanocrystal magnetic moments. According to the ACS analysis, the Brownian signal contribution corresponds to about 40% of the total signal and the rest is from Néel relaxation.

## 3. Experimental Section

Different nanoparticle systems have been prepared in organic and aqueous media, starting either from the co-precipitation of Fe(II) and Fe(III) salts, from a Fe(II) salt controlling oxidation, or from a Fe(III) salt controlling reduction. Finally, the particles have been surface-treated and coated with different materials such as silica, dextran or polystyrene, in order to avoid aggregation and achieve colloidal stability. Most of these methods of synthesis and coating have been described in a previous review [4].

CSIC01 particles were prepared by thermal decomposition of iron oleate in organic media and the subsequent silica coating by microemulsion. Particles used for CSIC04 and 5 samples were prepared by an oxidative precipitation process described before [16,17] starting from iron (II) sulfate and a basic solution containing sodium nitrate as mild oxidant. Then, a standard protocol was used to oxidize magnetite to maghemite (γ-Fe_2_O_3_) in acid media. These particles were dextran coated under high pressure homogenization (HPH) conditions and magnetically fractionated to obtain CSIC04 and CSIC05. Sample NPG3311 is a suspension of citrate-coated multi-core particles synthesized by aqueous co-precipitation of Fe(II) and Fe(III). SP02 sample consists of magnetic nanoparticles encapsulated in solid polymer spheres dispersed in water. It was prepared by a controlled precipitation process of the polymer that traps the nanoparticles in emulsion droplets by solvent evaporation.

The studied magnetic nanoparticle systems dispersed in water is stable for several months after synthetisation. This was determined by for instance using DLS and ACS methods that are sensitive to any possible particle aggregations in dispersed magnetic nanoparticle systems. However, we cannot exclude that no particle aggregation has taken place but the degree of aggregation (if any) does not change with time during the analysis phase of the synthesized particles.

Particle size distribution and shape were studied by TEM using a JEOL JEM-2000 FX microscope operated at 200 keV. The mean particle size and distributions were evaluated by measuring the largest internal dimension of at least 100 particles. Further analysis was performed using two different instruments: (1) a FEI Tecnai F20 equipped with a LaB6 electron gun and operated at 200 kV and (2) a FEI Titan 80–300 equipped with a field emission gun and operating at 80 or 300 kV. Sample preparations were carried out by placing a drop of diluted particle suspension on a Cu grid coated with a perforated carbon film, and leaving them to dry in air. The hydrodynamic size was studied using a DLS from Malvern Instrument to determine the hydrodynamic particle size in suspension. The dispersant was water and the temperature 25 °C.

Dynamic magnetization *vs*. excitation frequency (ACS) was performed with two AC susceptometers (DynoMag system and a lab AC susceptometer) and a high frequency susceptometer (lab AC susceptometer) in the frequency range from 1 to 10 MHz. These measurements were carried out at room temperature.

Magnetorelaxometry (MRX) measurements were performed with a setup utilizing a gradiometric fluxgate arrangement to record the stray field from the nanoparticle sample. MRX measurements are carried out for a magnetizing pulse of 2 mT amplitude and 2 s duration. Data analysis is restricted to time constants larger than 400 µs caused by the bandwidth of the fluxgate sensors and the switch-off time of the magnetization field. Measurements were all performed at room temperature.

## 4. Conclusions

We have shown that by adjusting the nanocrystal size and particle morphology, the dynamic magnetic properties can be changed both for single- and multi-core particles. We have also shown that it is possible to classify single- and multi-core magnetic particles by their magnetic relaxation properties and particle size parameters as determined by using dynamic magnetic analysis techniques from ACS and MRX analysis together with nanocrystal and hydrodynamic particle size information from TEM and DLS analysis.

We have also shown and described the objectives and activities in the EU FP7 NanoMag project that is focused on standardization of analysis methods for magnetic nanoparticles. In summary, the strategic and scientific objectives are listed below.

Strategic Objectives

- Identify analysis and characterization methods that can be used to standardize measurements of magnetic nanoparticles.
- Provide valuable tools in the manufacturing process of magnetic nanoparticles and when comparing results from different labs.
- Promote the standardization techniques for both research and industrial processes.
- Provide new metrological standards for magnetic nanoparticles.

Scientific Objectives

- Correlate magnetic and structural properties of magnetic nanoparticles.
- Develop new analysis methods and models for magnetic nanoparticles.
- Improve the ability to follow the whole life cycle of magnetic nanoparticle systems from synthesis stage to specific applications.

## References

[1] Pankhurst Q.A., Connolly J., Jones S.K., Dobson J. (2003). Applications of magnetic nanoparticles in biomedicine. J. Phys. D Appl. Phys..

[2] Krishnan K.M. (2010). Biomedical nanomagnetics: A spin through possibilities in imaging, diagnostics, and therapy. IEEE Trans. Magn..

[3] NanoMag-Project. www.nanomag-project.eu.

[4] Gutiérrez L., Costo R., Grüttner C., Westphal F., Gehrke N., Heinke D., Fornara A., Pankhurst Q.A., Johansson C., Veintemillas-Verdaguer S. (2015). Synthesis methods to prepare single- and multi-core iron oxide nanoparticles for biomedical applications. Dalton Trans..

[5] Ahrentorp F., Astalan A., Blomgren J., Jonasson C., Wetterskog E., Svedlindh P., Lak A., Ludwig F., van IJzendoorn L.J., Westphal F. (2015). Effective particle magnetic moment of multi-core particles. J. Magn. Magn. Mater..

[6] Ludwig F., Kazakova O., Barquín L.F., Fornara A., Trahms L., Steinhoff U., Svedlindh P., Wetterskog E., Pankhurst Q.A., Southern P. (2014). Magnetic, structural, and particle size analysis of single- and multi-core magnetic nanoparticles. IEEE Trans. Mag..

[7] Ahrentorp F., Astalan A.P., Jonasson C., Blomgren J., Qi B., Mefford O.T., Yan M., Courtois J., Berret J.F., Fresnais J. (2010). Sensitive high frequency AC suceptometry in magnetic nanoparticle applications. AIP Conf. Proc..

[8] Öisjöen F., Schneiderman J.F., Astalan A.P., Kalabukhov A., Johansson C., Winkler D. (2010). A new approach for bioassays based on frequency- and time-domain measurements of magnetic nanoparticles. Biosens. Bioelectron..

[9] Ferguson R.M., Khandhar A., Jonasson C., Blomgren J., Johansson C., Krishnan K.M. (2013). Size-dependent relaxation properties of monodisperse magnetite nanoparticles measured over seven decades of frequency by AC susceptometry. IEEE Trans. Magn..

[10] Chung S.H., Hoffmann A., Guslienko K., Bader S.D., Liu C., Kay B., Makowski L., Chen L. (2005). Biological sensing with magnetic nanoparticles using Brownian relaxation. J. Appl. Phys..

[11] Ludwig F. (2010). Characterization of Magnetic Core-Shell Nanoparticle Suspensions Using ac Susceptibility for Frequencies up to 1 MHz. AIP Conf. Proc..

[12] Ludwig F., Heim E., Schilling M. (2009). Characterization of magnetic core-shell nanoparticles by fluxgate magnetorelaxometry, ac susceptibility, transmission electron microscopy and photon correlation spectroscopy—A comparative study. J. Magn. Magn. Mater..

[13] Ludwig F., Mäuselein S., Heim E., Schilling M. (2005). Magnetorelaxometry of magnetic nanoparticles in magnetically unshielded environment utilizing a differential fluxgate arrangement. Rev. Sci. Instrum..

[14] Ludwig F., Remmer H., Kuhlmann C., Wawrzik T., Arami H., Ferguson R.M., Krishnan K.M. (2014). Self-consistent magnetic properties of magnetite tracers optimized for magnetic particle imaging measured by ac susceptometry, magnetorelaxometry and magnetic particle spectroscopy. J. Magn. Magn. Mater..

[15] Ludwig F., Guillaume A., Schilling M., Frickel N., Schmidt A.M. (2010). Determination of core and hydrodynamic size distributions of CoFe_2_O_4_ nanoparticle suspensions using ac susceptibility measurements. J. Appl. Phys..

[16] Andrés Vergés M., Costo R., Roca A.G., Marco J.F., Goya G.F., Serna C.J., Morales M.P. (2008). Uniform water stable magnetite nanoparticles with diameters around the monodomain—multidomain limit. J. Phys. D: Appl. Phys..

[17] Costo R., Bello V., Robic C., Port M., Marco J.F., Morales M.P., Veintemillas-Verdaguer S. (2012). Ultrasmall iron oxide nanoparticles for biomedical applications: Improving the colloidal and magnetic properties. Langmuir.

